# Pediatric Application of Cuffed Endotracheal Tube

**DOI:** 10.5811/westjem.59560

**Published:** 2023-04-28

**Authors:** Jung Heon Kim, Jung Hwan Ahn, Yun Jeong Chae

**Affiliations:** * Ajou University School of Medicine, Department of Emergency Medicine, Suwon, Korea; † Ajou University School of Medicine, Department of Anesthesiology, Suwon, Korea

## Abstract

A young child’s larynx was formerly believed to be narrowest at the cricoid level, circular in section, and funnel shaped. This supported the routine use of uncuffed endotracheal tubes (ETTs) in young children despite the benefits of cuffed ETTs, such as lower risk for air leakage and aspiration. In the late 1990s, evidence supporting the pediatric use of cuffed tubes emerged largely from anesthesiology studies, while some technical flaws of the tubes remained a concern. Since the 2000s, imaging-based studies have clarified laryngeal anatomy, revealing that it is narrowest at the glottis, elliptical in section, and cylindrical in shape. The update was contemporaneous with technical advances in the design, size, and material of cuffed tubes. The American Heart Association currently recommends the pediatric use of cuffed tubes. In this review, we present the rationale for using cuffed ETTs in young children based on our updated knowledge of pediatric anatomy and technical advances.

## INTRODUCTION

**Figure 1:**
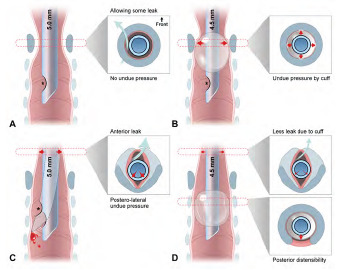
Schematic representation of shifts from uncuffed (A and C) to cuffed (B and D) endotracheal tubes, and from cricoid-circular-funnel (A and B) to glottis-elliptical-cylinder (C and D) laryngeal configuration. In addition, this schema depicts the myths 1 (A) and 2 (B) and recently discovered features (C and D). Each inset shows a transverse section with a tube shaft inserted at each level (marked in black). The cricoid cartilage is drawn as a blue-gray ring (insets in A and B) or V-shaped lamina (insets in C and D [upper]). B exemplifies an erroneously high cuff location caused by a Murphy eye (asterisks). C illustrates the posterolateral compression by the tube shaft. The compression is considered stronger than previously expected, given the shift in laryngeal configuration. C also shows that the tip of movable, uncuffed tube can injure the tracheal wall, which can be minimized by the added stability provided by a cuff. D depicts a high volume-low pressure cuff without a Murphy eye placed at an appropriate location, which results in stabilization of the tip by the cuff, less leak through the cuff, less pressure on the subglottis by the tube shaft (upper inset) and on the trachea by the cuff (lower inset). Airway injury may be further prevented by the posterior trachea, which distends when intracuff pressure increases. Numbers in millimeters indicate the inner diameters of the tubes.

The larynx of children younger than the age of eight (hereafter, “young children”) was thought to be narrowest at the cricoid level, circular in axial section, and funnel shaped. Thus, it was believed that the cricoid level was snugly fit by an uncuffed endotracheal tube (ETT) large enough to allow some air leakage around the tube at 20 centimeters of water (cmH_2_O) airway pressure (Figure 1A). In contrast, cuffed tubes incurred concerns of cuff-induced pressure exertion on the cricoid mucosa (Figure 1B), which can manifest as post-extubation stridor (PES) and potentially lead to subglottic stenosis. From this perspective, use of uncuffed tubes had been routinely favored for use in young children.^1^–^3^

Since 2003, imaging-based studies have clarified that the pediatric larynx is narrowest at the glottis, elliptical in section, and cylindrical in shape, like an adult larynx. This updated anatomic consideration coincided with a shift from the use of uncuffed to cuffed ETTs by anesthesiologists, which had already been initiated in the late 1990s (Figure 1). Initially, this shift was supported by the emerging benefits of cuffed tubes, chiefly cuff-induced adjustable sealing, which has been shown to result in less frequent tube changes (Table 1).^1^, ^2^, ^4^–^7^ Moreover, the shift was reinforced by contemporaneous technical advances such as high volume-low pressure (HVLP) polyurethane (PU) cuff.^8^ Currently, the American Heart Association (AHA) and the European Resuscitation Council recommend that young children be intubated with cuffed tubes.^9^,^10^

This topic has been discussed most commonly in the context of pediatric anesthesia or critical care.^2^, ^11^ However, there is a paucity of literature relevant to emergency department (ED) settings.^12^,^13^ This knowledge gap highlights the need to encourage the pediatric application of cuffed ETTs in ED practice. In this article, we review the literature addressing the use of cuffed tubes in young children based on the updated understanding of laryngeal anatomy and other rationales.

## METHODS

We searched PubMed and Scopus for articles in English using the keywords “intubation,” “cuffed,” and “child,” which had been published from 1997–2022. Of the searched items, we preferentially selected systematic reviews, narrative reviews, original articles, and editorials that describe the pediatric application of cuffed ETTs. Given the paucity of literature relevant to emergency settings, we had to include many articles authored by anesthesiologists. However, we excluded articles not focused on the benefits of cuffed tubes or the updated knowledge of the laryngeal anatomy in young children. We added manually searched articles regarding the updated laryngeal anatomy, other articles, textbooks, and guidelines. In total, this narrative review covered 66 articles (Supplement Figure 1), including three systematic reviews, two guidelines, four textbooks, 13 narrative reviews, seven randomized controlled trials, 12 experimental studies, 14 observational studies, four surveys, five editorials, one letter, and one case report.

## DISCUSSION

### Updated Laryngeal Anatomy: From the Cricoid-Circular-Funnel to the Glottis-Elliptical-Cylinder

The dogma of cricoid-circular-funnel shape was prevalent in pediatric practice due to a key article on infant laryngeal configuration that was based on autopsies showing the cricoid as the narrowest level in 15 children aged 4 months–14 years.^14^,^15^ A cadaveric glottis is more distensible than live human glottis owing to the laxity of devitalized tissue and the use of wax or plaster to fill up the larynx. In the autopsies, the glottis was probably overestimated relative to the circumferentially fixed cricoid.

**Figure 2:**
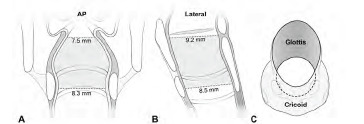
A laryngeal configuration based on the computed tomography-measured transverse diameters on AP view (A) and AP (ie, sagittal) diameters on lateral view (B).^18^ It is narrowest in the transverse diameter at the glottis (A). Looking down the larynx at 45° from above, elliptical section is noted at the glottis (C). The ellipticity means a potential for uncuffed tube-induced posterolateral compression (See Figure 1C). Modified from Kim et al.^20^ *AP*, anteroposterior.

Imaging-based studies on 86–401 live children have resulted in a revised understanding of pediatric laryngeal configuration from the cricoid-circular-funnel shape to the glottis-elliptical-cylinder shape (Table 2).^16^–^20^ The first refutation to the dogma came from Litman et al^16^ who measured laryngeal dimensions on magnetic resonance imaging. The measurement revealed a longer anteroposterior diameter than the transverse diameter (ie, elliptical), an increase in transverse diameter as we move caudad, and a linear association of age with the diameters at all levels. This means that the cylindrical larynx, with the glottis being the narrowest, grows proportionally without a configurational transition from the funnel to the cylinder. Subsequently, the implications have been confirmed by plain radiography, computed tomography (CT), and bronchoscopy.^17^–^20^ The CT-based studies proved differential sections per level: the more cephalad, the more elliptical (Figure 2).^18^–^20^

Holzki et al^21^,^22^ criticized the updated anatomy, proposing that movable vocal cords make the fixed cricoid the functionally narrowest laryngeal level and insisting that the cricoid is most prone to endoscopy-proven airway injury. This criticism is refuted by the following evidence: 1) autopsy reports show the narrowest level is at the glottis^21^,^23^; 2) the subglottis, which is less distensible than the glottis, has a smaller cross-sectional area (CSA) and volume than the cricoid^20^,^24^; 3) injury usually occurs in the posterolateral portions of the glottis or subglottis, relatively sparing the cricoid level^25^–^28^; and 4) the conus elasticus, a soft tissue extending from the lower border of the vocal cords to the upper border of the cricoid, is prone to edema in cases of intubation or croup, owing to its lax attachment.^20^,^28^,^29^ This feature makes the subglottis an obstruction-prone level. (5) On optical coherence tomography, airway wall thickness was correlated with intubation duration at the glottis and subglottis, not at the upper trachea.^27^ Hence, we speculate that some level between the glottis and subglottis is functionally narrowest in the larynx.

Briefly, the larynx in a young child is proportionally smaller than the adult larynx with the glottis or subglottis being the most injury-prone level. This update makes a valid rebuttal to the groundwork for the well-established use of uncuffed ETTs in young children.

### Myth Breakers: Benefits of Cuffed Tubes

The known benefits of cuffed ETTs involve lower risk for air leakage and aspiration around the cuffs, favoring their use in endotracheal intubation for older children and adults (Table 1).^1^,^2^ In young children, uncuffed tubes are often selected, whereas cuffed tubes were rarely used and restricted primarily to those with reduced lung compliance.^30^ The persistence of this choice was exemplified by a French survey in 1999 showing that only 25.4% of anesthesiologists used cuffed tubes in >80% of pediatric cases.^31^ At that time in EDs, cuffed tubes were probably used less frequently. This preference may have been affected by two myths derived from the false laryngeal configuration:

- Myth 1. Uncuffed tubes snugly fit the circular larynx (Figure 1A).
- Myth 2. Cuffs injure the cricoid mucosa (Figure 1B).

These myths were modified by the knowledge of the elliptical section of the larynx and the unexpectedly lower incidence of cuffed ETT-induced airway injury.

In a rebuttal to myth 1, a snugly fit, uncuffed ETT can incur ischemia by compressing the posterolateral mucosa, with a leak via anterior space (Figure 1C).^32^ To reduce such pressure, the tube should be relatively smaller in diameter than the snugly fitting size.^32^ This need can be met by using a cuffed tube, of which inner diameter (ID) is 0.5 millimeters (mm) smaller than a same age group-matched uncuffed tube (Figure 1D; cf, Cole and Duracher formulae in Supplement Table 1). If a cuffed tube is appropriately positioned, the tube shaft and cuff come in contact with the glottic-subglottic and tracheal mucosae, respectively. Thus, in the larynx, the relatively narrower tube shaft lowers risk for compression.

Contrary to myth 2, PES or other croup symptoms occur comparably in both types of ETTs (cuffed, 2.4%–4.4% vs uncuffed, 3.0%–4.7%).^33^,^34^ The occurrence of airway injury is associated not with the cuff per se, but with the following factors: intubation duration; tube size; traumatic intubation; intracuff pressure (P_cuff_); poorly designed or fit tube; movement of tube; low birth weight; infection; and shock.^5^,^11^,^30^,^35^ Further, sore throat more commonly occured with uncuffed tubes (cuffed, 7.7%–19.4% vs uncuffed, 32.4%–36.6%), indicating greater vulnerability to such minor injuries.^36^–^38^ This finding may be related to the contact of the tube tip with the tracheal wall, in addition to the posterolateral compression and frequent tube change mentioned above (Figure 1C).^2^,^34^,^39^,^40^ The tip-induced injury may deteriorate by movement of the tip during ventilation.^35^,^39^,^40^ If a cuffed tube is used, the cuff separates the tip and tracheal wall (Figure 1D).^32^–^34^;^39^,^40^

Additional benefits of cuffed ETTs need to be mentioned (Figure 1D). Two randomized controlled trials compared the two types of tubes in 488 (age ≤8 years) and 2,246 (≤5 years) anesthetized children, respectively.^33^,^34^ As per the trials, uncuffed tubes required more frequent changes (cuffed, 1.2%–2.1% vs uncuffed, 22.8%–30.8%).^33^,^34^ Moreover, the need for fewer cuffed tube changes was demonstrated by a 0.17 relative risk (95% confidence interval 0.07–0.41).^4^ This benefit may stem from the cuff volume, which is adjustable to seal the trachea when its diameter varies with airway pressure, sedation, muscle relaxation, or the patient’s position.^30^,^40^ This adjustability contrasts with the fixed outer diameter of uncuffed tubes.

To prevent cuff-induced tracheal injury, P_cuff_ should be limited to <20–25 cmH_2_O, since 20 cmH_2_O is presumed to be a capillary perfusion pressure in the tracheal mucosa.^9^,^41^ Theoretically, the posterior distensibility of the trachea may contribute to injury prevention (Figure 1D). Krishna et al^42^ showed 14, 23, and 45 cmH_2_O mean P_cuff_ of 5.0, 4.5, and 4.0 mm ID cuffed tubes, respectively, in a 10-mm ID, circumferentially fixed model trachea. In the tracheas of children aged 4–8 years, the mean P_cuff_ was 27 (5.0), 25 (4.5), and 31 cmH_2_O (4.0 mm).^42^ This slower increase in P_cuff_ in vivo indicates a pressure-buffering role of the posterior distensible trachea.

### Technical Flaws of Cuffed Tubes: Until the Early 2000s

Despite the benefits of cuffed tubes, concerns remained over their design, size, and material until the early 2000s. Compared to uncuffed tubes, cuffed tubes have an estimated 22%–52% margin of safety against intra-laryngeal cuff location and endobronchial intubation.^43^ Among the 11 cuffed tubes available in 2002, all cuffs of 3.0–5.0 mm ID tubes were located in the larynx with the tube tips at the mid-trachea.^44^ This erroneously high cuff location was related to the elongated shape of the cuff or the presence of distal Murphy eye (Figure 1B). Only five of the 11 products had depth marks, which should be leveled to the glottis to place the cuff below the cricoid. If a 3.0 mm ID tube was inserted with the mark at the glottis, three of the five products had their tips at the carina, indicating a too high location of the marks.^44^ Until the 1990s, a cuffed tube of size <5.0 mm ID was less available.^40^

Given the association between high P_cuff_ and airway injury, since the mid-1990s, HVLP cuffs have replaced high-pressure cuffs.^36^ With this change, there was increased clinical interest in studying to what degree high cuff volume is appropriate while limiting P_cuff_. At P_cuff_ of 20 cmH_2_O, CSA (or diameter) of the cuff should cover 120%–150% of CSA (or diameter) of the age group-related, maximally sized trachea.^1^,^45^ This high volume enables the cuff surface to drape along the tracheal wall, enhancing the sealing effect.^46^ As of 2002, most cuffs had CSAs that failed to meet the 120%–150% requirement.^44^ The 3.0–4.5 mm ID and 5.0–7.0 mm ID ETTs covered 71.4%–141.6% and 114.5%–301.0% of the tracheal CSAs, respectively.^44^ This indicates that the size was too small for children <5 years, and too large for older ones (Duracher’s, Supplement Table 1).^44^ A polyvinylchloride (PVC) cuff may create folds and channels on its surface, leading to leakage or airway injury.^1^ A 3.5–6.0 mm ID PVC cuffed tube (Mallinckrodt HiLo [Mallinckrodt Medical, Athlone, Ireland]) showed a median P_cuff_ of 23 cmH_2_O (maximum, 120 cmH_2_O) with only 40.8% of P_cuff_ <20 cmH_2_O.^47^

### Contemporaneous Technical Advances in Cuffed Tubes

PU emerged as an HVLP cuff material while conventional PVC was still being used. Advances in the design, size, and material of cuffed tubes is represented by the Microcuff^TM^ (Microcuff GmbH, Weinheim, Germany), a PU-cuffed ETT released in 2004. This product features a short, distally located, cylindrical, 10 micrometer (μm)-thick cuff (cf, PVC, 50–80 μm), absence of Murphy eye, properly located depth mark, and a size ranging from 3.0–7.0 mm ID (for children weighing ≥3.0 kilograms [kg]).^1^,^8^,^48^,^49^ The PU cuff enabled sealing with a mean P_cuff_ of 9.7 cmH_2_O with 1.6% and 1.8% oversize and PES rates, respectively.^8^

PU is a better cuff material than PVC in meeting the 120%–150% requirement of HVLP cuffs and maintaining low P_cuff_. Fischer et al^50^ compared two PU cuffs (Microcuff and Parker ThinCuff PTCL [Parker Medical, Danbury, CT]) and three PVC cuffs of 3.0–7.0 mm ID tubes at 20 cmH_2_O P_cuff_, in terms of sealing the age group-related maximally sized tracheas. As a result, the PU and PVC cuffs covered 110%–129% and 68%–157% of the tracheal diameters, respectively. Of note, the PVC cuffs of 3.0–4.5 mm ID tubes tended to insufficiently seal the trachea (68%–114%). A study comparing one PU cuff (Microcuff) and three PVC cuffs of 4.0 mm ID tubes in 80 children 2–4 years old showed a median P_cuff_ of 11 cmH_2_O in the PU cuff, in contrast to 21–36 cmH_2_O in the PVC cuffs.^51^ Compared to PVC cuffs, PU cuffs have a smaller difference between measured and manufacturer-provided cuff diameters, and expand more symmetrically.^50^ Compared to PVC cuffs, ultrathin PU cuffs result in fewer or finer folds and channels, preventing leakage and aspiration.^49^,^52^ Consistent with the benefits of cuffed tubes and the updated anatomy, technical advances in cuff tube design have facilitated their application in young children.

### Current Recommendations for Cuffed Tubes

Cuffed ETTs have gained popularity in anesthesia worldwide. Approximately 70%–90% of Dutch and 50%–80% of British anesthesiologists preferred cuffed tubes for children aged 1 month–8 years.^53^ Another survey showed that using the tubes in ≥50% of occasions for those with the same age range was reported in 74%–85% of the Society of Pediatric Anesthesia members, of whom 88% were from the United States.^54^ These proportions contrast with the 25.4% of anesthesiologists surveyed in 1999.^31^ As of 2019, in an academic hospital in Maryland, it was decided to discontinue use of uncuffed tubes in the operating rooms.^55^

The current guidelines are consistent with the updated anatomy and technical advances, promoting the pediatric application of cuffed tubes in EDs. The 2020 American Heart Association recommendation for use of cuffed ETTs facilitates the translation of the tubes from operating rooms into emergency departments.^9^ In addition, cuffed tubes are recommended for children—except “small” infants—by the 2021 European Resuscitation Council guidelines.^10^ The most recent emergency medicine textbooks recommend cuffed tubes or at least highlight their benefits, whereas a representative textbook of pediatrics does not discuss the topic (Supplement Table 2 lists textbook descriptions).^56^–^59^

### Is the Anesthesiologic Evidence Applicable to EDs?

Unlike elective intubation under anesthesia, emergency intubation features urgency, lack of nil per os, greater frequency of crash airways, shorter length of induction, longer intubation duration, and variable skill levels of intubators. In EDs, critically ill or injured children should be stabilized with first-pass success of intubation and positive pressure ventilation. Cuffed tubes require fewer tube changes due to the adjustability of the cuffs. Even if a tube smaller than the best fitting size is intubated (ie, undersized intubation), which leads to excessive leakage at 20 cmH_2_O P_cuff_, a cuffed tube expedites positive pressure ventilation by temporarily hyperinflating the cuff and permitting high P_cuff_, or vice versa, permitting some leakage around the cuff.^12^ After stabilization, it may be replaced with a larger tube. Undeniably, airway resistance could rise more acutely in a 0.5 mm ID smaller cuffed tube than in an uncuffed tube.^42^ Such an issue can be eased by applying pressure-controlled ventilation or, if spontaneous ventilation is possible, pressure-support ventilation.^6^ Essentially, uncuffed tubes require more frequent tube changes as compared with cuffed tubes. If undersized, uncuffed tubes more easily develop an unacceptable degree of leakage or aspiration, incurring inaccurate delivery of tidal volume or occurrence of ventilator-associated pneumonia.^7^,^37^

With increased awareness of pediatric laryngeal anatomy and technical advances, cuffed ETTs are becoming the norm for emergency intubation in young children.^9^,^10^ Hence, we recommend the preferential use of cuffed tubes in EDs while awaiting ED-based evidence.

### Three Caveats

First, it is recommended to monitor P_cuff_ of <20–25 cmH_2_O using a cuff manometer (Table 1). Although the monitoring is associated with a reduction of PES from 21.8% to 9.9%,^60^ a cuff manometer is rarely available in EDs. Instead, many emergency physicians slowly inflate cuffs until the cessation of audible leakage around the cuffs, despite the unreliability of this maneuver.^56^ Compared to the maneuver, P_cuff_ estimation by palpating the cuffs is related to even higher P_cuff_.^61^ As an interim measure in the case of the unavailability of a manometer, it may be acceptable to slowly put 0.5 milliliters (mL) of air (maximum 1 mL) using a 1-mL syringe until the leaking stops. This maneuver is supported by 0.6 mL of air required to achieve 20 cmH_2_O P_cuff_ of a 3.0 mm ID Microcuff tube in a model trachea and the association between 0.9 mL median air volume and 12 cmH_2_O median P_cuff_ in 44 children with a median age of three years.^62^,^63^

Second, small-sized ETTs (eg, <5.0 mm ID) need more judicious cuff inflation and size estimation, or the use of PU cuffs. If a formula is used, we recommend the Duracher formula instead of Khine’s (Supplement Table 1).^62^ In children weighing ≥3.0 kg, the small size of cuffed tubes might lead to inadvertent undersize, inducing inevitable rises in airway resistance and P_cuff_. This scenario is plausible given the association of a 0.5 mm decrease in the ID of tubes with higher mean P_cuff_ (Khine-estimated, 25 cmH_2_O vs 0.5 mm smaller tube, 37 cmH_2_O),^42^ and more frequent PES, hoarseness or sore throat if estimated by Khine’s than by Duracher’s formula.^62^ Undersized intubation may predispose children to an obstruction by mucus plugging or if bronchoscopy is required, a need for tube change to a larger size.

Third, in neonates or infants weighing <3.0 kg, it remains prudent to use uncuffed ETTs. In this population, a 3.0 mm ID cuffed tube may still be too large for their airways and cause airway injuries more frequently than an uncuffed tube. The injury is more likely to occur when cuffed tubes are inserted into infants with low birth weight or the tracheal wall is in contact with the wrinkled edge of a deflated cuff.^64^,^65^ In those infants weighing 2–3 kg, cuffed tubes may be chosen in >50% of occasions at ≥2.7 kg weight.^11^,^66^ Reportedly, a 2.5 mm ID Mircocuff tube is currently under development.^11^

## LIMITATIONS

First, there might have been a potential exclusion of articles mentioning the pediatric difficult or crash airway situations during the exclusion process of searched articles. Despite the insufficient evidence, we speculate that the use of cuffed ETTs may be beneficial in those situations. Second, the impact of sedatives or neuromuscular blocking agents on leakage or aspiration was not detailed given that regardless of the choice between cuffed and uncuffed tubes, the drugs are used during rapid sequence intubation or critical care.

## CONCLUSION

A young child’s larynx features the glottis as the narrowest level, elliptical section, and cylindrical shape. This updated anatomic consideration and technical advances are facilitating the use of HVLP cuffed ETTs, particularly tubes with PU cuffs. In emergency intubation of young children, cuffed tubes are preferred to uncuffed tubes while monitoring low P_cuff_, judiciously inflating cuffs of small-size tubes, and continuing to use uncuffed tubes in neonates or infants weighing <3.0 kg.

## Supplementary Information





## Figures and Tables

**Table 1:** Comparative benefits and limitations of cuffed endotracheal tubes over uncuffed tubes.^1^,^2^,^4^–^7^,^33^,^34^

Variable	Feature*	Remark
Emerging benefits†	Improved seal and less need for tube change	Cuff size is adjustable to variable tracheal sizes at same age
	Adjustable fit	Lower rate of oversized intubation
	Similar incidence of severe injury (eg, PES)	Cuffed, 2.4%–4.4% vs uncuffed, 3.0%–4.7%
	Lower incidence of minor injury (eg, sore throat)‡	Tube shaft-induced posterolateral compression of the glottis-subglottis
		Cuff-induced separation of tube tip and the trachea prevents tracheal injury
Established benefits§	Less leakage	More reliable delivery/monitoring of tidal volume/capnography
		Less consumption of/pollution by anesthetics
	Less aspiration	Lower rate of ventilator-associated pneumonia
Limitations	Need for intracuff pressure monitoring†	Safe range: <20–25 cmH_2_O (ideally, using cuff manometer)
		0.5–1.0 mL of air may be sufficient to inflate cuffs of 3.0–5.0 mm ID tubes
	Available down to size 3.0 mm ID	Still recommended to use uncuffed tubes in <3 kg neonates
	Higher airway resistance due to 0.5 mm-smaller ID	Compensated by pressure-support ventilation
		Difficult suctioning
	Higher cost	Compensated by less need for tube change and more reliable ventilation

* Listed in the order of relevance in emergency settings, rather than of frequency.

† The benefits have become known since the mid-1990s. Although the benefits are of cuffed tubes per se, they have been reinforced, and a lower intracuff pressure is enabled by the use of high volume-low pressure, polyurethane cuffs.

‡ Refer to Figure 1C.

§ Known before the mid-1990s and thereafter, accumulation of relevant evidence.

*ID*, inner diameter; *PES*, post-extubation stridor; *cmH_2_O*, centimeters of water; *mL*, milliliter; *kg*, kilogram; *mm*, millimeter.

**Table 2:** Literature on imaging-based, updated understanding of laryngeal anatomy.^16^–^20^

Author	Study design and setting	Narrowest dimension	AP-to-transverse ratio	Association/correlation of diameter with age
Litman et al (2003)^16^	N = 99, 2 mo-13 y (mean, 61.6 mo), MRI under PSA, and 1 center in the United States	Transverse glottic diameter*	>1 at all levels†	Linear association in all diameters at all levels
Dalal et al (2009)^17^	N = 128, 6 mo-13 y (mean, 70.8 mo), bronchoscopy under anesthesia/paralysis, and 2 centers in the U.S.	Transverse glottic diameter CSA: 30.0 mm^2^ (glottis) vs. 48.9 mm^2^ (cricoid)	>1 at all levels†	Linear association in CSA at all levels
Wani et al (2016)^18^	N = 130, 1 mo-10 y (mean, 47.4 mo), CT under PSA, and 1 center in Saudi Arabia	Transverse glottic diameter CSA: 55.9 mm^2^ (subglottis) vs. 57.1 mm^2^ (cricoid)	1.2 at the subglottis‡ 1.0 at the cricoid‡	Correlation in all diameters at all levels
Mizuguchi et al (2019)^19^	N = 86, 1 mo-15 y (median, 53 mo), CT ± PSA, and 1 center in Japan	Transverse subglottic diameter	1.5 at the subglottis§ 1.1 at the cricoid§	Correlation in transverse glottic diameter
Kim et al (2022)^20^	N = 401, 1 mo-4 y (median, 26.0 mo), plain radiography, and 1 center in Korea	Transverse glottic diameter* CSA: 26.5 mm^2^ (glottis) vs. 40.5 mm^2^ (cricoid)	2.9 at the glottis‡ 1.1 at the cricoid‡	Correlation in all diameters at all levels

* In the two studies, the glottis and subglottis were defined separately. Otherwise, the two levels were defined interchangeably.

† Unavailable detailed numerical data.

‡ Calculated with the reported mean or median values.

§ The ratios remained generally constant per age group.

*MRI*, magnetic resonance imaging; *PSA*, procedural sedation and analgesia; *CT*, computed tomography; *CSA*, cross-sectional area; *AP*, anteroposterior; *mm^2^*, square millimeter.
