# Supplementary material for: Pediatric Application of Cuffed Endotracheal Tube
Source: West J Emerg Med. 2023 Apr 28;24(3):579–87. doi: 10.5811/westjem.59560 (PMC10284523; doi:10.5811/westjem.59560)
Supplement: Supplementary file 1 [file wjem-24-579-s001.pptx]

## Slide 1
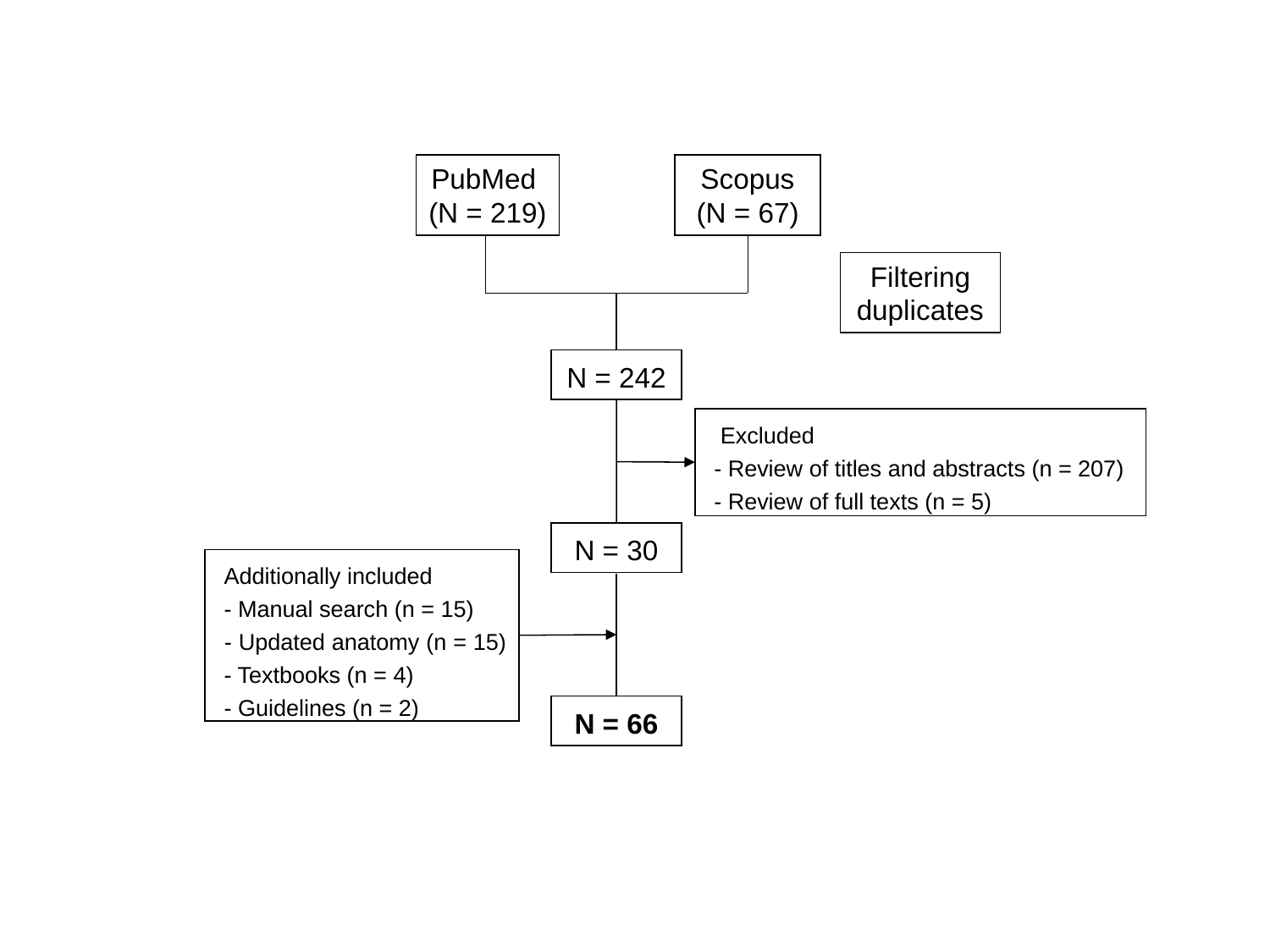

PubMed
(N = 219)
Scopus
(N = 67)
Filtering duplicates
N = 242
 Excluded
 - Review of titles and abstracts (n = 207)
 - Review of full texts (n = 5)
N = 30
 Additionally included
 - Manual search (n = 15)
 - Updated anatomy (n = 15)
 - Textbooks (n = 4)
 - Guidelines (n = 2)
N = 66
