# Supplementary material for: Pediatric Application of Cuffed Endotracheal Tube
Source: West J Emerg Med. 2023 Apr 28;24(3):579–87. doi: 10.5811/westjem.59560 (PMC10284523; doi:10.5811/westjem.59560)
Supplement: Supplementary file 2 [file wjem-24-579-s002.docx]

**Supplement Table 1.** Age-based formulae for estimation of endotracheal tube size

| Common portion | Eponyms | | |
| --- | --- | --- | --- |
|  | Cole | Duracher^*^ | Khine |
| $Tube size (mm)=R+\frac{Age (y)}{4}$ | *R* = 4.0 | *R* = 3.5^†^ | *R* = 3.0^†^ |
|  | Uncuffed | Cuffed, preferred | Cuffed |

^*^Also known as Motoyama.

^†^Smaller constants are applied to compensate for cuff-occupying space.

**Supplement Table 2.** Descriptions regarding the choice between cuffed and uncuffed ETTs in textbooks of EM or pediatrics

| Textbook | Description |
| --- | --- |
| Tintinalli’s EM ninth ed, 2019 | “It is generally recommended that cuffed ETTs be used down to size 3.5, which would typically be the size for a full-term newborn.” |
| Rosen’s EM ninth ed, 2018 | “Current cuff technology can accurately measure cuff inflation pressures, and cuffed tubes may be preferred, particularly in instances of high airway pressures or poor compliance (eg, asthma, pneumonia, and acute respiratory distress syndrome). Utilizing a cuffed ETT may obviate the need to replace and upsize a tube when there is significant air leak that impacts ventilation.” |
| Fleisher and Ludwig’s textbook of pediatric EM eighth ed, 2021 | “Pediatric Advanced Life Support guidelines as well as the anesthesia literature now support that, beyond the newborn period, cuffed ETTs are equally as safe as uncuffed tubes.” |
| Nelson’s textbook of pediatrics 21th ed, 2020 | No specific mention on the topic. |

ETT, endotracheal tube; EM, emergency medicine.
